# Comparison and characterization of enriched mesenchymal stem cells obtained by the repeated filtration of autologous bone marrow through porous biomaterials

**DOI:** 10.1186/s12967-019-02131-y

**Published:** 2019-11-19

**Authors:** Wenxiang Chu, Yifu Zhuang, Yaokai Gan, Xin Wang, Tingting Tang, Kerong Dai

**Affiliations:** 1 Shanghai Key Laboratory of Orthopaedic Implants, Department of Orthopaedic Surgery, Shanghai Ninth People’s Hospital, Shanghai Jiao Tong University School of Medicine, Shanghai, 200011 China; 2 Department of Orthopedic Surgery, Changzheng Hospital, Second Military Medical University, Shanghai, 200003 China

**Keywords:** Bone marrow, Mesenchymal stem cell, Enrichment technique, Porous biomaterial, Filtration parameter

## Abstract

**Background:**

When bone marrow is repeatedly filtered through porous material, the mesenchymal stem cells (MSCs) in the bone marrow can adhere to the outer and inner walls of the carrier material to become enriched locally, and this is a promising method for MSC enrichment. In this process, the enrichment efficiency of MSCs involved in the regulation of the cell ecology of postfiltration composites containing other bone marrow components is affected by many factors. This study compared the enrichment efficiency and characterized the phenotypes of enriched MSCs obtained by the filtration of autologous bone marrow through different porous bone substitutes.

**Methods:**

Human bone marrow was filtered through representative porous materials, and different factors affecting MSC enrichment efficiency were evaluated. The soluble proteins and MSC phenotypes in the bone marrow before and after filtration were also compared.

**Results:**

The enrichment efficiency of the MSCs found in gelatin sponges was 96.1% ± 3.4%, which was higher than that of MSCs found in allogeneic bone (72.5% ± 7.6%) and porous β-TCP particles (61.4% ± 5.4%). A filtration frequency of 5–6 and a bone marrow/material volume ratio of 2 achieved the best enrichment efficiency for MSCs. A high-throughput antibody microarray indicated that the soluble proteins were mostly filtered out and remained in the flow through fluid, whereas a small number of proteins were abundantly (> 50%) enriched in the biomaterial. In terms of the phenotypic characteristics of the MSCs, including the cell aspect ratio, osteogenetic fate, specific antigens, gene expression profile, cell cycle stage, and apoptosis rate, no significant changes were found before or after filtration.

**Conclusion:**

When autologous bone marrow is rapidly filtered through porous bone substitutes, the optimal enrichment efficiency of MSCs can be attained by the rational selection of the type of carrier material, the bone marrow/carrier material volume ratio, and the filtration frequency. The enrichment of bone marrow MSCs occurs during filtration, during which the soluble proteins in the bone marrow are also absorbed to a certain extent. This filtration enrichment technique does not affect the phenotype of the MSCs and thus may provide a safe alternative method for MSC enrichment.

## Background

Among bone repair materials, autologous bone grafts have been considered the gold standard owing to the three major properties of osteoconductivity, osteoinductivity, and osteogenesis [1, 2]. Although some bone substitute materials characterized by osteoconductivity or osteoinductivity (such as porous bioceramics, decalcified bone matrix, and allogeneic bone) have certain bone repair abilities, it is difficult for them to play their assigned roles without osteogenic progenitor cells [3]. The lack of osteogenic progenitor cells in the implanted region is a limiting factor that compromises the bone repair effects of such materials [4–7]. Therefore, the integration of osteogenic progenitor cells with existing bone substitutes can allow the biomaterials to mimic autologous bone in terms of its composition and bone repair abilities. Bone tissue engineering can utilize this strategy. Although the bone repair effect is reliable, the approach used to obtain sufficient seed cells by in vitro culturing is accompanied by several drawbacks, including long culturing times, expensive procedures and equipment, high costs, and the ethical controversy related to the use of animal serum, all of which make this approach difficult to implement in clinical practice.

In autologous bone, the osteogenic progenitor cells attached to the trabecular bone are derived from the bone marrow, which is an important source of precursor cells of osteogenic progenitor cells, including mesenchymal stem cells (MSCs) [8, 9]. If the living environment of autologous bone could be mimicked to allow the direct mixing of bone marrow with porous bone substitute material and to cause the MSCs in the bone marrow to adhere to the surface of the porous material, composites of specific numbers of MSCs with porous materials could be formed without in vitro culturing. Unfortunately, MSCs only account for 0.001–0.01% of bone marrow nucleated cells [10, 11]. To acquire sufficient MSCs without culturing, the absolute number of MSCs has to be inevitably increased by raising the bone marrow volume. This highlights the importance of the effective combination of MSCs derived from large amounts of bone marrow with biomaterials.

Mesenchymal stem cell enrichment technology focuses on techniques for isolating sufficient amounts of MSCs using nonculturing methods. Presently, the primary methods of MSC enrichment include density gradient centrifugation and the use of a screen-enrich-combine circulating system for MSCs [12–16]. The former method obtains a bone marrow nucleated cell layer rich in MSCs via density gradient centrifugation of the bone marrow [12, 17]. By using a porous material as a filtration medium, the latter method, at the time of filtration, utilizes the quick adhesion of MSCs to the inner and outer surfaces of porous materials during filtration because of the rapid adhesion characteristic of MSCs [13, 14]. Consequently, the cell-material combination is directly achieved. This filtration enrichment method does not depend on expensive instruments, and three steps (cell screening, enrichment, and the combination of cells with materials) can be quickly completed with one process during surgery, which suggests the enormous potential of this method for clinical application. Nevertheless, throughout the filtration process, the combination of bone marrow cells with materials is influenced by multiple factors, including the selection of carrier materials, the volume of bone marrow used for filtration, and the appropriate filtration time and circulating frequency. When these influencing factors remain unclear, it is rather difficult to ensure the efficient enrichment of MSCs. A low cell enrichment efficiency suggests that MSCs and other components in a large amount of bone marrow could not be fully utilized, thereby reducing the osteogenic ability of the composite material and leading to the inefficient use of bone marrow from patients. Therefore, specific factors that critically influence the enrichment efficiency of bone marrow cells during filtration and enrichment are of great significance for the clinical application of enrichment techniques. In addition, the investigation of the specific components enriched within the carrier material after filtration and the assessment of the effect of filtration on cell safety have important clinical value for implants.

In this study, we primarily focused on the following three issues to comprehensively evaluate the feasibility and safety of constructing composite osteogenic materials by utilizing bone marrow MSCs via autologous bone marrow filtration through porous bone substitutes: (1) specific factors affecting cell enrichment efficiency, including the differences among carrier materials, the bone marrow/material volume ratio, and the filtration frequency; (2) the main components of the composite after filtration and their relative quantitative distribution; and (3) the potential impact of filtration on cell safety.

## Materials and methods

### Collection and distribution of bone marrow

This study was reviewed by the ethics committee of the Shanghai Ninth People’s Hospital, Shanghai Jiao Tong University School of Medicine. All volunteers were fully informed and signed informed consent forms before participation.

A total of 23 adult volunteers underwent bone marrow collection. For each volunteer, bone marrow (less than 30 ml) was taken from multiple sites (4 ml/site) in the anterior superior iliac spine and mixed well with heparin (100 U/ml). Bone marrow collection and distribution are described in

**Table 1:** Distribution of bone marrow aspiration

Groups	Number of volunteers	Collecting volume	Volume for filtration	Volume for detection
Types of carrier materials	9	21 ml	20 ml	1 ml
Volume ratio of bone marrow/carrier material	3	29 ml	4/10/14 ml	1 ml
Filtration frequency	5	21 ml	20 ml	1 ml
Components and safety evaluation	6	22 or 24 ml	20 ml	1 + 1 + 2 ml^a^

^a^1 ml for counting of bone marrow nucleated cells and MSCs (six volunteers); 1 ml for primary bone marrow MSCs culture (six volunteers); 2 ml for bone marrow serum extraction to detecting soluble protein in bone marrow (one volunteer)

**Fig. 1:**
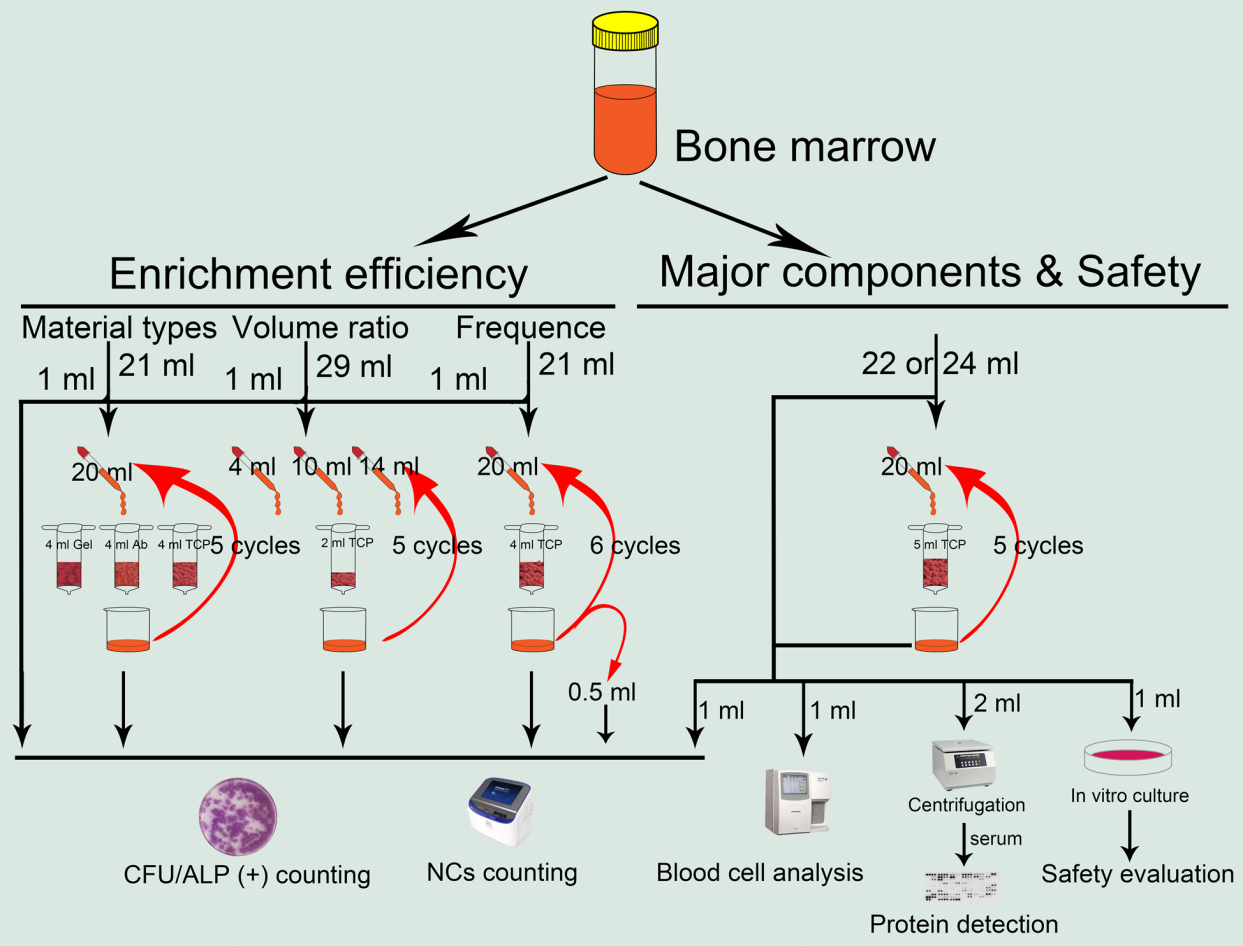
Schematic diagram of the experimental procedure

Table 1, and schematic illustrations of all the methods of MSC enrichment used are shown in Fig. 1.

### Selection and treatment of carrier materials

The carrier materials were selected among porous biological materials (commonly used in clinical practice) to meet the requirements of the filtration of bone marrow. They comprised allogeneic cancellous bone particles, porous β-tricalcium phosphate (β-TCP) particles and porous absorbable gelatin sponges. Prior to use, the allogeneic femoral head provided by Shanghai Anjiu Biotechnology Co., Ltd. was placed into a bone crusher (Germany) and ground into granulated particles (3–5 mm in diameter). The absorbable gelatin sponges (State Food and Drug Administration Approval number: H32024096, Nanjing, China) were cut into 5 mm × 5 mm granules. The porous β-TCP particles (3–5 mm in diameter; 75% ± 10% porosity; 500 ± 150 μm pore diameter; interconnection diameter 150 ± 50 μm; interconnected pores > 99%) were purchased from Shanghai Biolu Biomaterials Co., Ltd.

### Bone marrow filtration and evaluation of the specific factors affecting the efficiency of the enrichment of bone marrow cells

1. The carrier material was placed in a 10 ml syringe, from which the bone marrow was filtered at a flow rate of 20 ml/min. The bone marrow samples before and after filtration were used for counting and analyzing the enrichment efficiency of nucleated cells (NCs) and MSCs. (1) To explore the influence of different materials on the enrichment efficiency of bone marrow cells, 20 ml of bone marrow was filtered 5 times with each carrier material (4 ml). (2) To study the influence of the bone marrow/carrier material volume ratio on the bone marrow cell enrichment efficiency, 4 ml (twice), 10 ml (5 times), and 14 ml (7 times) of bone marrow were filtered 5 times with 2 ml of porous β-TCP particles. (3) To investigate the influence of the filtration frequency on the cell enrichment efficiency, 20 ml of bone marrow was filtered 6 times through 4 ml of porous β-TCP particles. The bone marrow was thoroughly mixed after each filtration, and 0.5 ml was used for NC and MSC counting.
2. To analyze the cell components and soluble protein components in the filtered carrier materials and to evaluate the cell safety of the filtration process, 20 ml of bone marrow was filtered 5 times through 5 ml of β-TCP particles, and the bone marrow samples before and after filtration were used for blood cell analysis, MSC culturing, and bone marrow serum isolation before and after filtration.

### NC and MSC counting and enrichment efficiency calculation

The bone marrow samples before and after filtration were centrifuged, and the supernatants were removed. The samples were then thoroughly mixed with red blood cell lysate (TBD, China), and the obtained NCs were diluted twofold with complete media (alpha-MEM containing 10% [v/v] fetal bovine serum). The diluted cells were resuspended; 10 μl of cell suspension was aspirated and mixed with 10 μl of trypan blue dye solution. Ten microliters of the mixed suspension was evaluated using an instrument (Countess^®^ II, Thermofisher) for the NC counting and cell viability assays. The cell count results were multiplied by two for correction to determine the original number of bone marrow NCs. The number of MSCs was assessed according to the number of alkaline phosphatase-positive cell colonies. Before and after filtration, the bone marrow samples were diluted in complete media and inoculated into a 6-well plate (0.1 ml bone marrow/well). The cells were cultured in a 5% CO_2_ incubator at 37 °C, and the media was changed every 2–3 days. When cell colonies appeared, the cell culture media was replaced with osteogenic induction media. After 7 days of induction, alkaline phosphatase staining was performed, and the number of alkaline phosphatase-positive colony-forming units (CFU/ALP+) was counted. The enrichment efficiency was calculated according to the formula

$$ {\text{E}}\, = ({\text{N}}_{\text{pre}} - {\text{N}}_{\text{post}} )/{\text{N}}_{\text{pre}} \times 100\% , $$

where N_pre_ and N_post_, respectively, represent the number of NCs and MSCs per ml of bone marrow before and after filtration.

### The evaluation of the cell number distribution in the postfiltration composite biomaterial

The relative quantitative distribution of various components in the material was evaluated by measuring the changes in bone marrow composition before and after filtration. In this study, the number of red blood cells (RBCs), white blood cells (WBCs), and platelets (PLs) in the bone marrow before and after filtration were determined by blood cell analysis. The cell components within the filtered material were calculated according to the formula

$$ {\text{N}} = {\text{N}}_{\text{pre}} \times {\text{V}}_{\text{pre}} - {\text{N}}_{\text{post}} \times {\text{V}}_{\text{post}} , $$

where N_pre_ and N_post_ represent the number of cells (including RBCs, WBCs, PLs, and MSCs) per ml of bone marrow before and after filtration, respectively. V_pre_ and V_post_ refer to the total volume of bone marrow before and after filtration, respectively.

To compare the relationship between the relative quantity of MSCs with that of other types of cells in the postfiltration composite biomaterial and the original bone marrow, the numbers of other types of cells were normalized to the number of MSCs by calculating the ratio of N_Cells_/N_MSCs_ (N_Cells_ represents the total number of RBCs, WBCs or PLs in the composite biomaterial after filtration, and N_MSCs_ indicates the total number of MSCs in the material). Similarly, the ratio of the two in the original bone marrow was calculated.

### Observation of bone marrow cells in the postfiltration composite biomaterial

The freshly filtered composites and the composites after 2 weeks of in vitro culture were used to observe the cells within the composites. Before observation by a scanning electron microscope, the samples were sequentially washed with PBS, fixed with glutaraldehyde, dried at critical points, and subjected to metal spraying. After 2 weeks of culture, the composites were fixed with 4% paraformaldehyde and incubated with 0.5% Triton-100 for 5 min. Then, rhodamine and DAPI were used to stain the cell cytoskeleton and nuclei, respectively. Afterwards, the samples were observed under a confocal laser scanning microscope (CLSM, Leica TSC SP8, Germany).

### The evaluation of the soluble protein components in composite biomaterials after filtration

For determining the soluble protein components, 1.5 ml of bone marrow from a volunteer was centrifuged at 300*g* for 5 min before and after filtration, and the bone marrow serum was extracted. The high-throughput, semiquantitative analysis of the cytokine content in bone marrow serum was performed using the Human XL Cytokine Array Kit (ARY022B, Univ, China). Grayscale values were used to indicate the results of the semiquantitative analysis. The absorption efficiency of the soluble proteins obtained from the filtration process was calculated with the formula

$$ {{\text{A}}}_{\text{protein}} = ({{\text{D}}}_{\text{pre}} - {\text{D}}_{\text{post}})/ {\text{D}}_{\text{pre}} \times 100 \%, $$

A_protein_ refers to the absorption efficiency and D_pre_ and D_post_ represent the grayscale values of the soluble proteins before and after filtration, respectively.

### Primary and secondary culture of cells for the in vitro identification of MSCs

One milliliter of bone marrow was thoroughly mixed with 10 ml of complete media. The cell mixture solution was then inoculated into a 10 cm petri dish and cultured in a 5% CO_2_ incubator at 37 °C. Three days later, the media was changed, and it was replaced every 2 days. When the spindle cells fully covered the bottom of the Petri dish, secondary culture was performed. Specifically, the media were aspirated, and the cells were washed twice with phosphate-buffered saline (PBS). The cells were incubated with 0.05% trypsin at 37 °C for 2 min, followed by the addition of serum-containing complete medium to neutralize the trypsin. The detached cells were gently pipetted and collected into a 50 ml centrifuge tube. After centrifugation, the cells were resuspended in complete media and inoculated into new culture dishes.

### Comparison of MSC morphology

The aspect ratio of the adherent MSCs was evaluated. The primary cell images were taken after culturing the cells for 10 days before and after filtration. For each group, 15 cells in the peripheral region of the cell colony that clearly presented an intact morphology of an individual cell were used for the aspect ratio measurement.

### Comparison of the osteogenetic differentiation fate of MSCs before and after filtration

Mesenchymal stem cells before and after filtration were stimulated with osteogenic medium (Cyagen, USA) at 37 °C in 5% CO_2_ for 7, 14 and 21 days, respectively. After 7 days of stimulation, alkaline phosphatase expression (ALP) and activity were measured. In brief, the cells were washed with PBS and immersed in ALP dye solution for 40 min to observe the expression of ALP after being fixed with 4% paraformaldehyde for 20 min. The activity of ALP was detected using an ALP microplate test kit (Nanjing Jiancheng Bioengineering Institute, China) following the manufacturer’s instructions. At 14 days, immunofluorescence staining of osteopontin (OPN) were performed. After the samples were fixed with 4% paraformaldehyde, they were sequentially incubated with a specific primary antibody against OPN (ABCAM) and 488-AffiniPure anti-rabbit Ig-G. The cytoskeleton and nuclei were also stained with rhodamine and DAPI, respectively, before observation by CLSM. After 21 days of induction, alizarin red staining and quantification were carried out as described in a previous report [18].

### MSC phenotype identification

The first generation of MSCs before and after filtration were selected for cell phenotype identification and comparison. The antibodies used included anti-CD34-PE, anti-CD45-PE, anti-CD90-PE, and anti-CD105-APC (BD Bioscience; USA). Antibodies were not added to the blank control, and the corresponding isotype controls were simultaneously used with the positive surface markers. The cell suspension and the antibody solution were incubated on ice for 60 min in the dark. Subsequent to the centrifugation of the mixture solution, the cells were washed 3 times with 0.5% BSA/PBS and resuspended, followed by analysis using flow cytometry.

### Analysis of the MSC cycle

When the first generation of cells before and after filtration were cultured to 85% confluence, the cells were washed 3 times with PBS and trypsinized to prepare the cell pellets. The cell pellets were washed with 30 ml of PBS, which was removed after centrifugation at 1000 rpm for 10 min. The cells were fixed with a fixative solution at 4 °C for 20 min. The fixative solution was then removed, and the cell pellets were vortexed, after which 5 ml of precooled 75% alcohol was added and incubated with the cells for 3 h at − 20 °C. Following this, the cells were washed successively with PBS and staining solution to remove the residual alcohol. The supernatant was removed after centrifugation, and the cells were resuspended in 0.5 ml of PI/RNase staining buffer and incubated for 15 min at room temperature. Flow cytometry analysis was performed within 1 h.

### Apoptosis assay of MSCs

Once the primary cells were cultured to 85% confluence before and after filtration, cell apoptosis was evaluated. The specific steps were performed according to the manufacturer’s instructions. Briefly, the cells were washed twice with cold PBS, and a 1 × 10^6^/ml cell suspension was made in 1× binding buffer. The cell suspension (100 μl) was placed in a 2 ml culture tube, and 5 μl of annexin V-FITC and 5 μl of PI were added. The mixture solution was mixed gently and allowed to stand at room temperature for 15 min. Subsequently, 400 μl of 1× binding buffer was added, and the results were measured by flow cytometry within 1 h.

### MSC gene expression analysis

Once the primary cells had reached 85% confluence before and after filtration, 1000 μl of TRIzol was added, and the cells were repeatedly pipetted until they were fully dissolved in the TRIzol, followed by storage at − 80 °C. A gene chip (Oebiotech, China) was used to detect the differences in gene expression in primary cells before and after filtration. Three samples (from the same volunteer) before and after filtration were used for the replication analysis.

### Data statistics

The data analysis was performed using SPSS 24.0 software (IBM, Armonk, New York, USA). The data used were expressed as the mean ± standard deviation. Student’s t-test was used to compare the mean values between the two groups, and the paired data were analyzed by a paired t-test. The mean values of multiple groups of data were compared using one-way analysis of variance. The differences between groups were analyzed using the least significant difference t-test. *P* < 0.05 was considered statistically significant.

## Results

### Factors affecting the cell enrichment efficiency during filtration

**Fig. 2:**
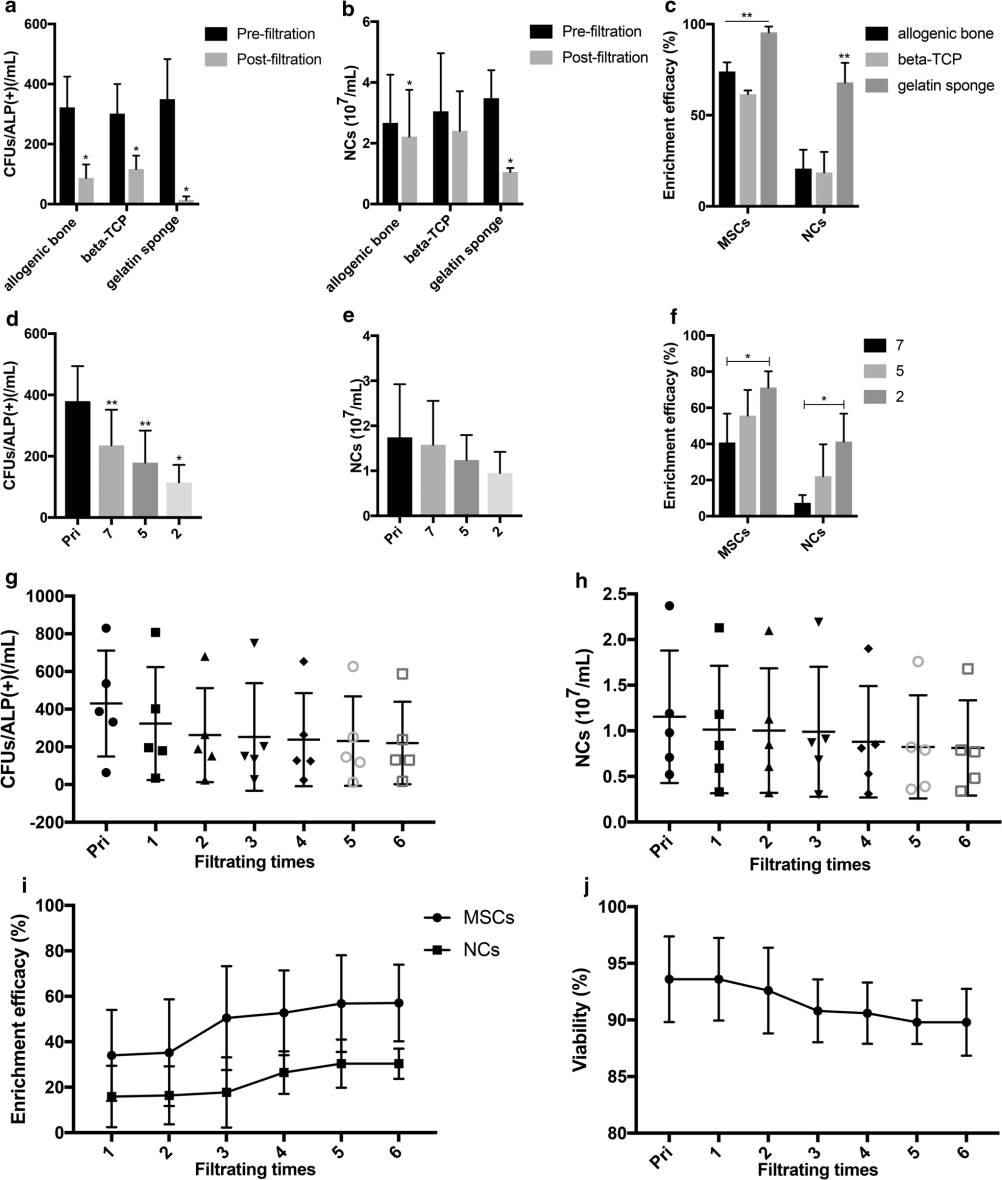
Specific factors influencing the cell enrichment efficiency during filtration. **a**–**c** Changes in the numbers of MSCs and NCs in bone marrow and their respective enrichment efficiencies after filtration through different materials (allogeneic bone particles, β-TCP, and gelatin sponges); **d**–**f** when the volume ratio of the bone marrow/carrier material was 7, 5, and 2; the differences in the numbers of bone marrow MSCs and NCs after filtration in terms of enrichment efficiency; **g**–**i** the increase in filtration frequency, the changes in the numbers of bone marrow MSCs and NCs, and their respective enrichment efficiency; **j** the impact of the filtration frequency on the viability of bone marrow cells

After filtration through three types of carrier materials, the number of MSCs in bone marrow was significantly decreased. The number of NCs in bone marrow had also declined to some extent (Fig. 2a, b). The enrichment efficiency of MSCs was as follows: gelatin sponge (96.1% ± 3.4%) > allogeneic bone particles (72.5% ± 7.6%) > porous β-TCP particles (61.4% ± 5.4%) (F = 66.480, P = 0.000). The NC enrichment efficiency was as follows: gelatin sponge (68.0% ± 10.8%) > allogeneic bone particles (20.7% ± 10.3%) and β-TCP particles (18.6% ± 11.3%) (F = 20.129, P = 0.002) (Fig. 2c).

The volume of the material was constant, and the change in the blood volume of the filtered bone marrow affected the cell enrichment efficiency. The number of MSCs in the bone marrow before filtration was 380.0 ± 114.2/ml on average and decreased to 114.7 ± 57.5/ml (t = 7.397, P = 0.018), 179.3 ± 104.2/ml (t = 20.958, P = 0.002) and 235.7 ± 116.2/ml (t = 12.356, P = 0.006), respectively, after filtration with bone marrow with a volume that was two-, five-, and sevenfold the volume of the carrier material); the nucleated cell reduction did not show significant differences at these three ratios (Fig. 2d, e). After bone marrow filtration at the aforementioned three ratios, the enrichment efficiency of the bone marrow MSCs was 71.2% ± 8.9%, 55.6% ± 14.2% and 40.8% ± 15.9%, respectively; the efficiency was significantly higher at a ratio of 2:1 than at a ratio of 7:1 (P = 0.032). The nucleated cell enrichment efficiency was 41.3% ± 15.5%, 22.1% ± 17.6% and 7.4% ± 4.3%, respectively, and a significantly higher enrichment efficiency was observed at a ratio of 2:1 than at a ratio of 7:1 (P = 0.032) (Fig. 2f).

Along with the increase in filtration frequency, the number of MSCs in bone marrow generally tended to decrease, and the enrichment efficiency was gradually increased and became stable after 5–6 filtrations (Fig. 2g–i). The NCs also exhibited a similar trend, but their enrichment efficiency was consistently lower than that of MSCs (Fig. 2h, i). However, as the frequency of filtration increased, the enrichment efficiency increased, and the cell viability was affected at the same time (Fig. 2j). After performing six filtrations, the cell activity remained at 89.8% ± 2.9%.

### Identification of the major components in the composite material after filtration

**Fig. 3:**
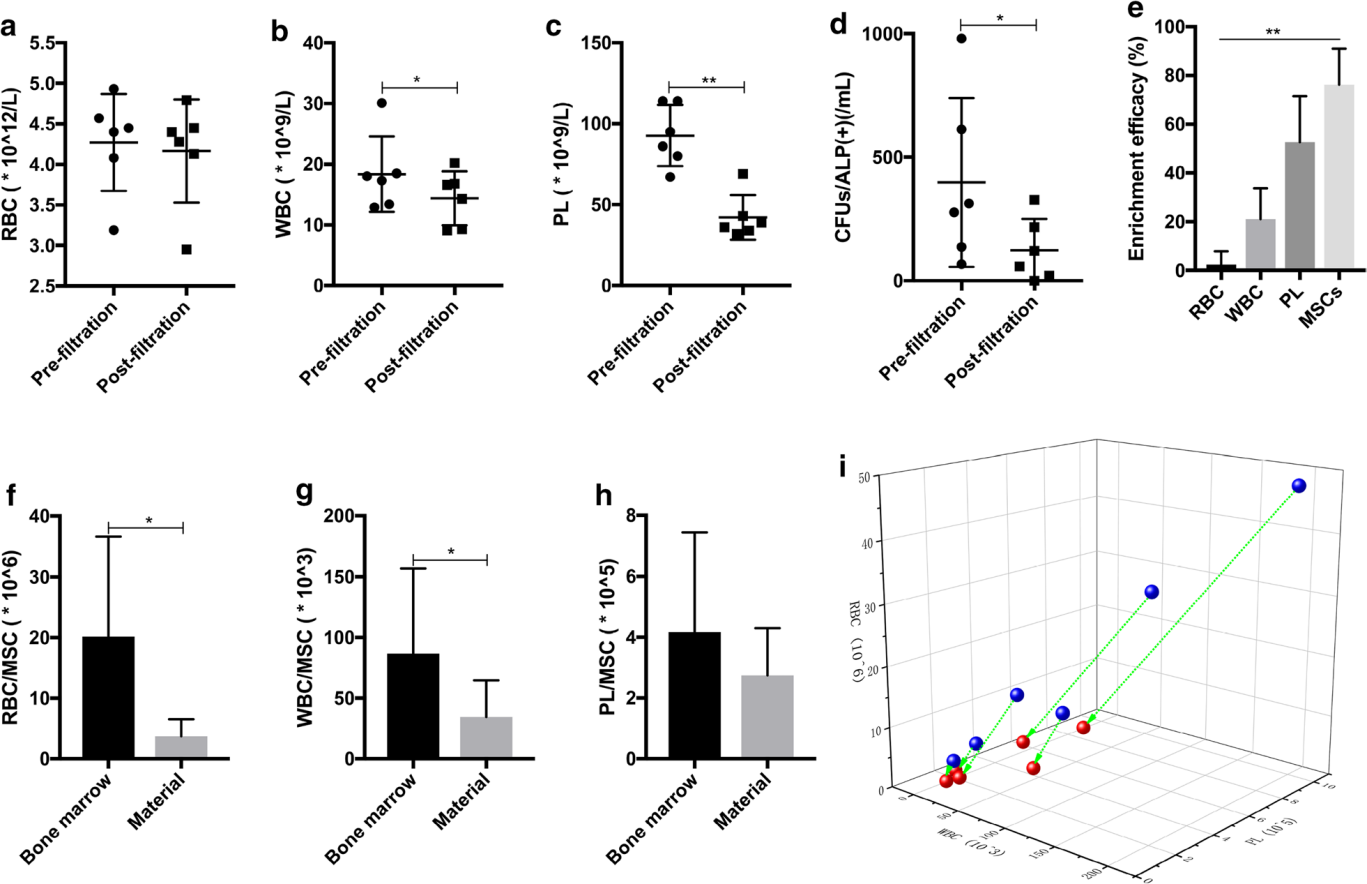
Changes in the numbers of different types of cells before and after filtration and the quantitative distribution of these cells in the composite. **a**–**d** Changes in the number of RBCs, WBCs, PLs, and MSCs (CFU/ALP+) in bone marrow before and after filtration; **e** the enrichment efficiency of RBCs, WBCs, PLs, and MSCs after filtration; **f**–**h** the difference in the numbers of other types of cells (RBCs, WBCs, or PLs) normalized to that of the respective MSCs in the original bone marrow and postfiltration composite biomaterial; **i** the arrow-connected blue and red balls, respectively, represent the quantitative distribution of RBCs, WBCs, and PLs corresponding with each MSC in original bone marrow (blue balls) and in the filtered composite material (red balls) from the same volunteer

The results of the bone marrow cell analysis before and after filtration revealed changes in the numbers of WBCs (18.37 ± 6.22 × 10^9^/l vs 14.38 ± 4.44 × 10^9^/l; t = 2.974, P = 0.031) and PLs (92.67 ± 18.86 × 10^9^/l vs 42.17 ± 13.70 × 10^9^/l; t = 4.954, P = 0.004). However, RBCs showed no significant changes (4.27 ± 0.60 × 10^12^/l vs 4.17 ± 0.64 × 10^12^/l; t =1.128, P = 0.310) (Fig. 3a–c). The in vitro CFU/ALP+ comparison showed a significant reduction in MSCs (379.83 ± 341.91/ml vs 123.67 ± 126.82/ml; t = 3.094; P = 0.027) (Fig. 3d). The following results were obtained for the enrichment efficiency: MSCs (76.23% ± 14.81%) > PLs (52.56% ± 18.93%) > WBCs (21.05% ± 12.71%) > RBCs (2.51% ± 5.40%) (F = 33.522, P = 0.000) (Fig. 3e).

The primary cell components in the material after filtration were RBCs (13.04 ± 1.98 × 10^9^), WBCs (116.78 ± 73.14 × 10^6^), PLs (1123.38 ± 457.05 × 10^6^), and MSCs (6801.27 ± 5307.55). For each individual MSC, the material had fewer RBCs (3.42 ± 3.00 × 10^4^ vs 20.19 ± 16.44 × 10^4^; t = −2.957, P = 0.032) and WBCs (30.81 ± 28.28 × 10^3^ vs 86.87 ± 70.08 × 10^3^; t = −2.778, P = 0.039) than that in the original bone marrow, while no significant differences were noted for PLs (248.01 ± 163.78 × 10^3^ vs 416.70 ± 327.34 × 10^3^; t = −1.717, P = 0.147), suggesting that filtration may have a high screening capability for MSCs and PLs (Fig. 3f–i). Figure 3i clearly presents the changes in other cell components after MSC calibration during filtration. Specifically, the numbers of RBCs and WBCs associated with each MSC in the filtered material were significantly decreased compared with that in the original bone marrow. Since PLs and MSCs had a tendency to be simultaneously enriched within the material, the ratio of PLs to MSCs was not significantly different from that in the original bone marrow.

**Fig. 4:**
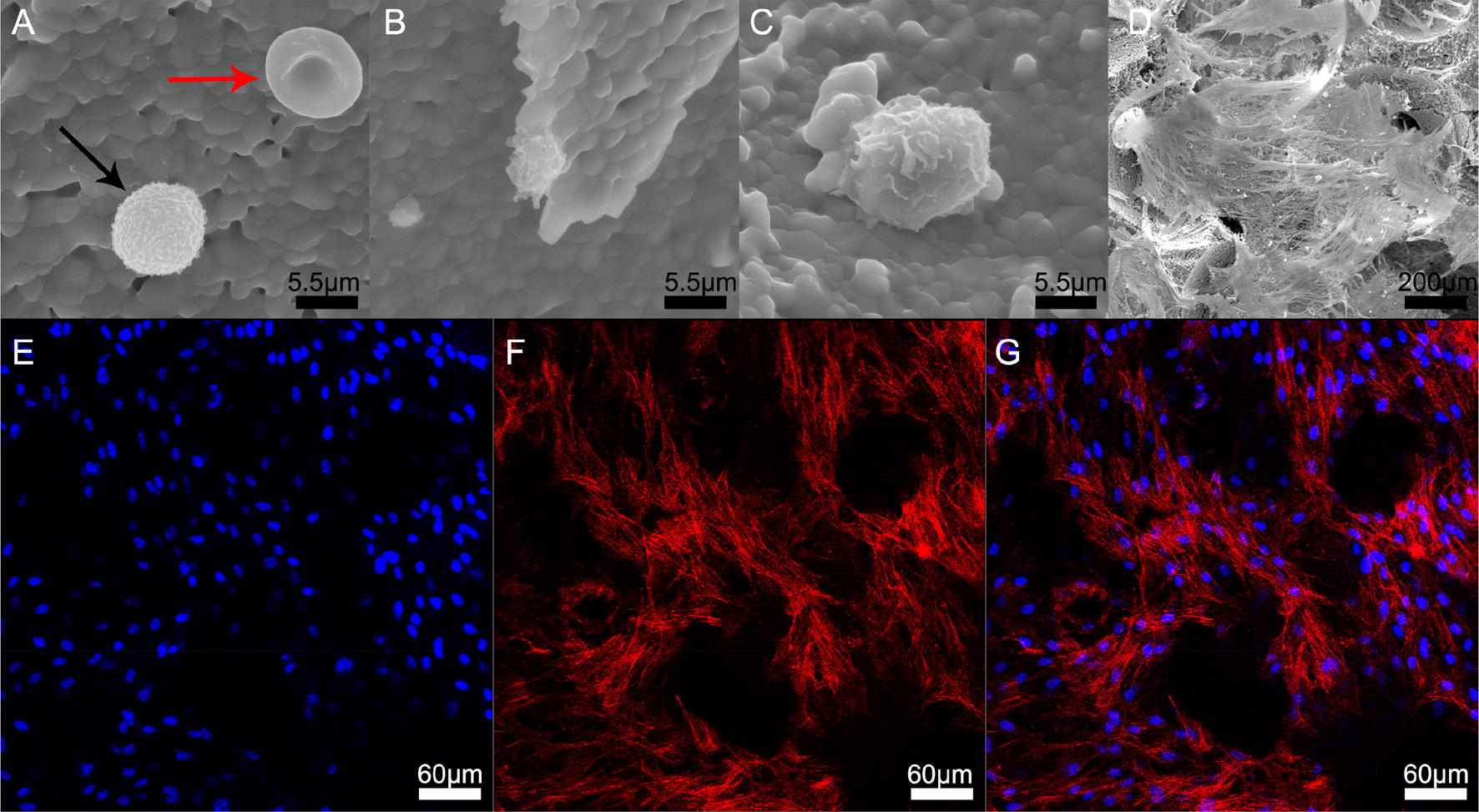
Observation of cellular components in the composite material after filtration. **A**–**D** After filtration, the composite material was incubated for 4 h and then subjected to electron microscopy. Spheroid WBCs (black arrow, **A**), disc-shaped RBCs (red arrow, **A**), activated PLs (**B**) with protruding pseudopods, and MSCs (**C**) were observed. **D**–**G** After 2 weeks of culture, electron microscopy (**D**) and confocal microscopy (**E**–**G**) showed that the cells were widely distributed and fully spread in the material

To observe the cellular components in the filtered material more directly, electron microscopy was performed on the composite material immediately after filtration (Fig. 4). The results showed that the interior surface of the material was covered with spheroidal WBCs, discoid RBCs, and activated PLs and MSCs full of pseudopods. After 2 weeks of in vitro culturing, the imaging results of the electron microscopy and confocal microscopy showed that the MSCs had proliferated and spread extensively inside the material.

**Fig. 5:**
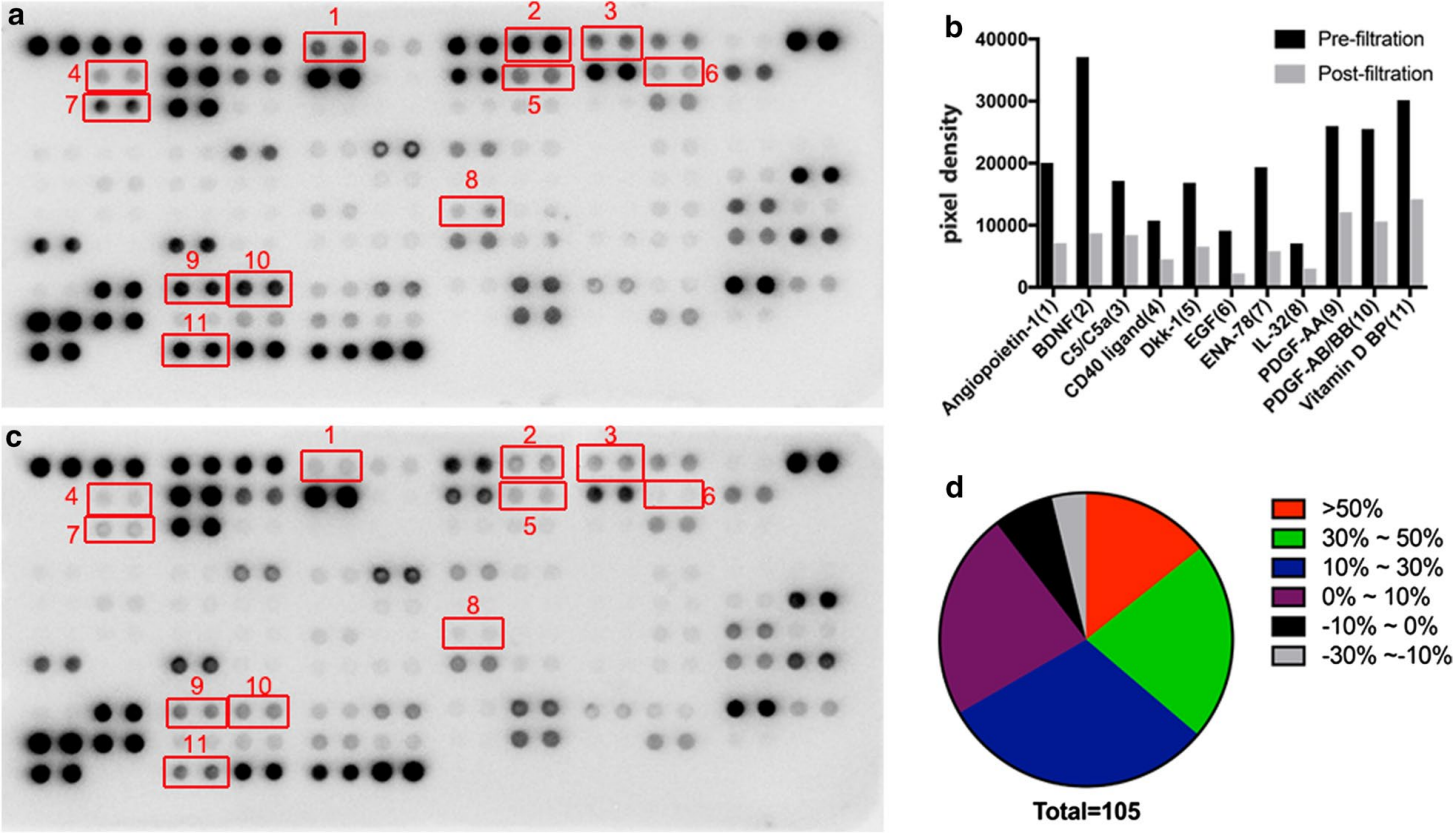
Absorption of active factors from the bone marrow by the carrier material during filtration. **a**–**c** The active factors in the original bone marrow serum (**a**) and in the postfiltration bone marrow serum (**c**). The active factors in the original bone marrow with a grayscale value that was greater than 10% of the reference value and was reduced by more than 50% after filtration are circled in the red box and listed in (**b**); **d** the proportions of active factors with different postfiltration absorption rates among the total tested active factors

The high-throughput antibody microarray results revealed the distribution of soluble proteins in bone marrow before and after filtration (Fig. 5a–c). The proteins with grayscale values that were more than 10% of the reference value and had decreased by more than 50% after filtration are listed in Fig. 5b. Overall, more than half of the original amount of few (15/105) of the active factors were absorbed by the carrier material after filtration (Fig. 5d), and most of them were directly filtered along with the fluid.

### Evaluation of the influence of filtration on cell safety

**Fig. 6:**
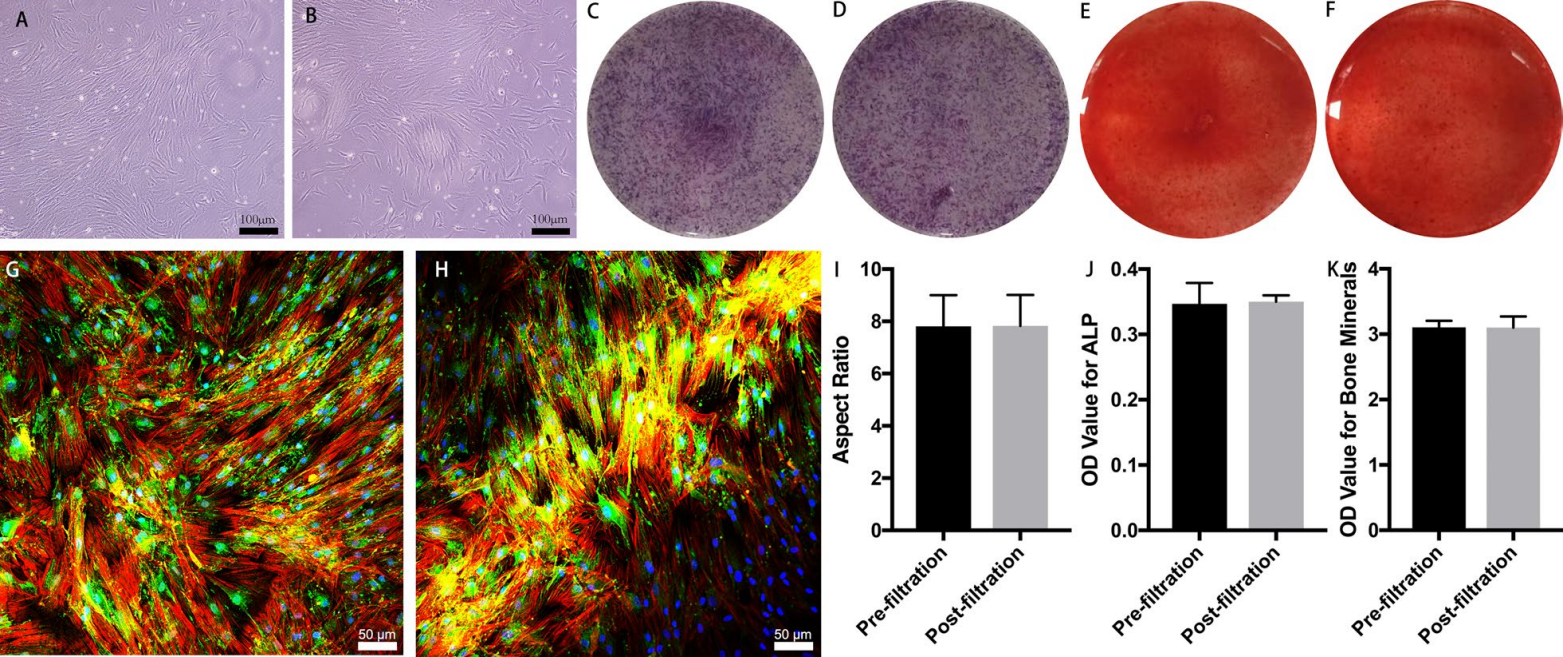
Comparison of MSC morphology and osteogenetic differentiation fate before and after filtration. **A**, **B** Primary bone marrow MSCs were cultured for 10 days before (**A**) and after filtration (**B**), and the cell morphology was observed on day 10; **C**, **D** Alkaline phosphatase staining of pre- (**C**) and postfiltration (**D**) MSCs and their quantitative analysis (**J**). **E**, **F** Alizarin red staining of pre- (**E**) and postfiltration (**F**) MSCs and their quantitative analysis (**K**). **G**, **H** The osteogenetic marker OPN was stained green in pre- (**G**) and postfiltration (**H**) MSCs. **I** No significant difference was noted in the MSC aspect ratio before and after filtration; *OPN* osteopontin

**Fig. 7:**
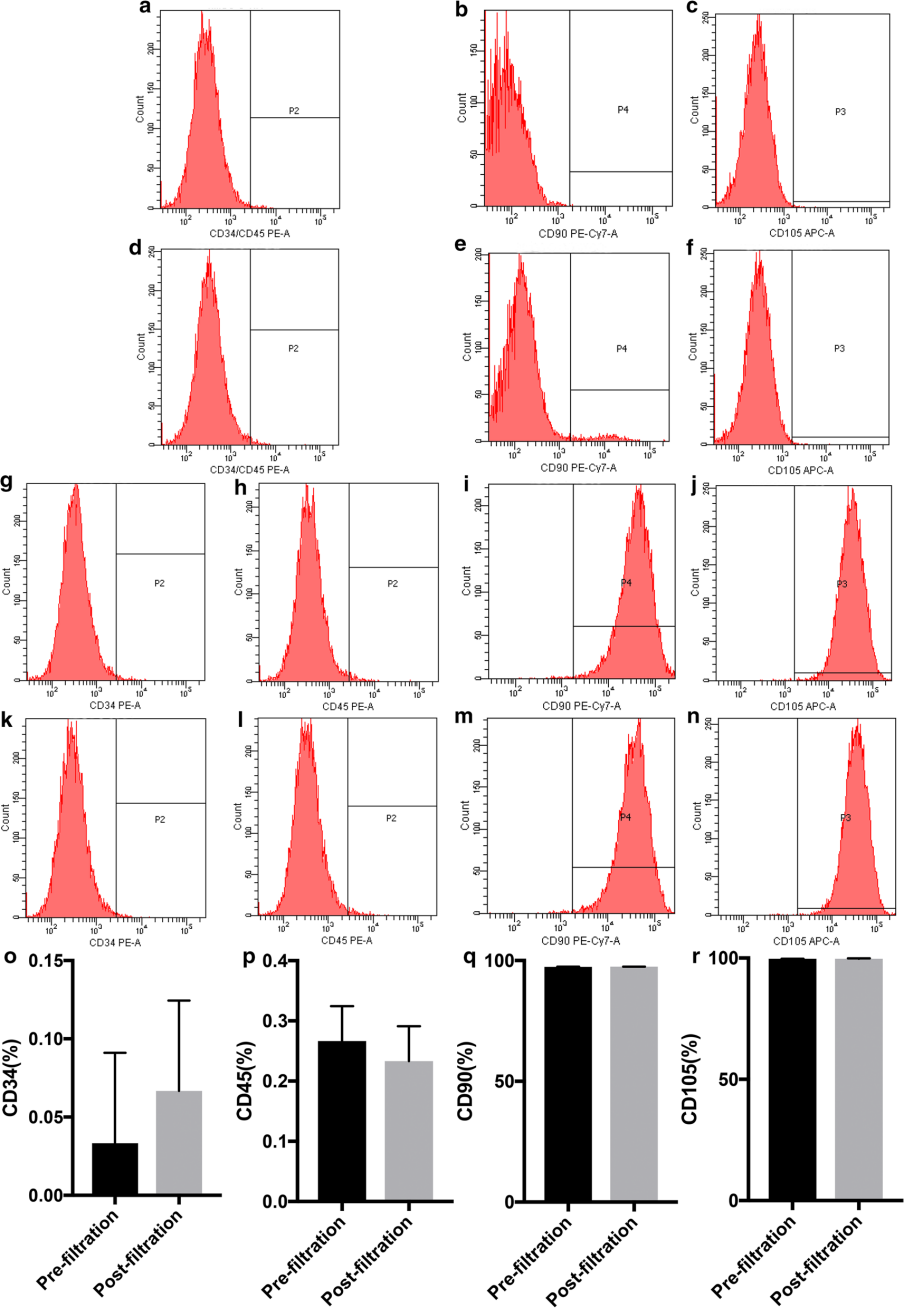
Comparison of the surface molecular markers in first passage of MSCs before and after filtration. **a**–**c** Negative control; **d**–**f** isotype control; **g**–**j** cell surface molecular markers before filtration; **k**–**n** cell surface molecular markers after filtration; **o**–**r** quantitative comparison of cell surface molecular marker expression before and after filtration

**Fig. 8:**
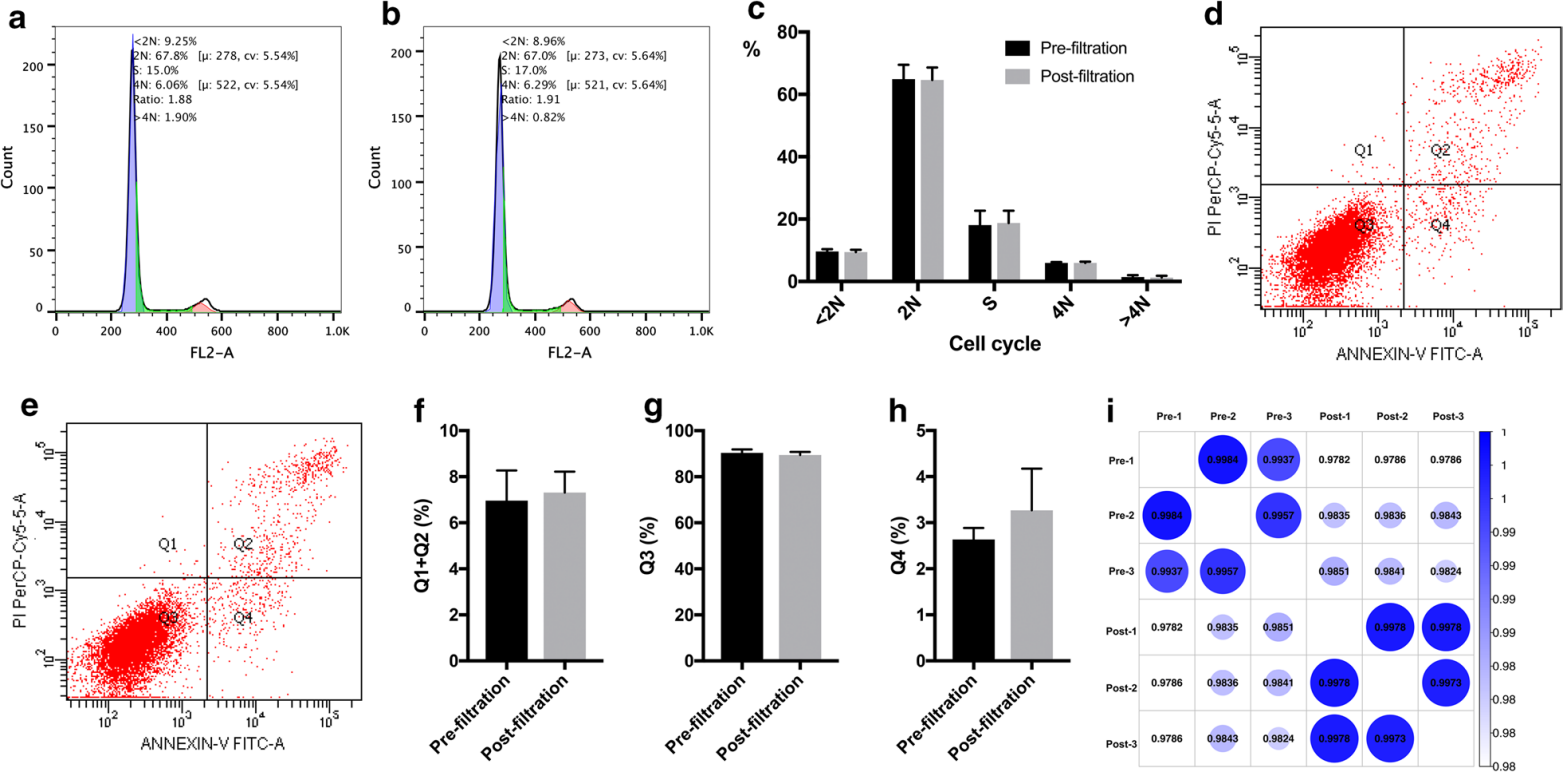
Comparison of the cell cycle, apoptosis and the gene expression profile in MSCs before and after filtration. **a**, **b** The cell cycle of MSCs isolated before filtration (**a**) and after filtration (**b**) in bone marrow with a cell cycle overlap of 85%; **c** quantitative comparison of the MSC cell cycle stages before and after filtration. **d**, **e** Comparison of the apoptosis of MSCs extracted from bone marrow before filtration (**d**) and after filtration (**e**) with a cell cycle overlap of 85%; **f**–**h** quantitative comparison of the proportions of MSCs in various apoptotic stages before and after filtration. **i** Comparison of the gene expression profile similarities of primary MSCs extracted from bone marrow before and after filtration. Pre-1, pre-2, and pre-3 represent the three replicates of primary bone marrow MSCs donated by the same volunteer before filtration; Post-1, post-2, and post-3 represent the three replicates of primary bone marrow MSCs from the volunteer donor after filtration

The influence of filtration on cell safety was evaluated using MSCs in bone marrow. No significant difference was noted in the cell aspect ratio before and after filtration (7.82 ± 1.19 vs 1.83 ± 1.18; t = −0.037, P = 0.971) (Fig. 6A, B, I). Filtration did not inhibit the express of ALP (Fig. 6C, D, J), the bone mineralization (Fig. 6E, F, K) and osteogenic marker of OPN expression (Fig. 6G, H) of MSCs that differentiated toward osteogenesis. No significant change was noted in the expression of cell surface markers, including CD90 (97.4% ± 0.1% vs 97.4% ± 0.1%; t = 1.414, P = 0.230), CD105 (99.7% ± 0.1% vs 99.7% ± 0.1%; t = 0.894, P = 0.422), CD34 (0.1% ± 0.1% vs 0.0% ± 0.1%; t = 0.707, P = 0.519), and CD45 (0.2% ± 0.1% vs 0.3% ± 0.1%; t = −0.707, P = 0.519) (Fig. 7a–r). In addition, the cell cycles of simultaneously cultured MSCs were also highly consistent before and after filtration. When the cell overlapping rate reached 85%, similar numbers of < 2N (9.6% ± 0.8% vs 9.4% ± 0.7%; t = 0.292, P = 0.785), 2N (64.9% ± 4.5% vs 64.6% ± 4.0%; t = 0.076, P = 0.943), S (18.1% ± 4.6% vs 18.8% ± 4.0%; t = −0.199, P = 0.852), 4N (6.0% ± 0.3% vs 6.0% ± 0.4%; t = −0.051, P = 0.962), and > 4N (1.4% ± 0.6% vs 1.2% ± 0.6%; t = 0.447, P = 0.678) cells were observed (Fig. 8a–c). Similarly, when simultaneously cultured primary MSCs grew to 85% confluence, the cell apoptosis status was essentially the same. The proportions of dead and late apoptotic cells (7.0% ± 1.3% vs 7.3% ± 0.9%; t = −1.429, P = 0.289), early apoptotic cells (2.6% ± 0.3% vs 3.3% ± 0.9%; t = −0.965, P = 0.437), and normal cells (90.4% ± 1.5% vs 89.5% ± 1.4%; t = 1.197, P = 0.354) were measured. The results presented in Fig. 8d–h show that mechanical stimulation in the enrichment process did not increase the probability of MSC apoptosis in the bone marrow during the subsequent proliferation processes. In addition, the gene expression profiles of the MSCs showed high similarity before and after filtration, and only 5.3% (1070/20,030) (Fig. 8i) of the expressed genes were altered to some degree.

## Discussion

Important goals in the field of orthopedic research have been to develop bone repair materials with improved osteogenetic capability, osteoinductivity, and osteoconductivity and to become less dependent on the use of autologous bones [19, 20]. Because MSCs play indispensable roles in bone repair, several cell-processing strategies have been used for MSC extraction and their combination with traditional bone repair materials to enhance their osteogenic capacity [4, 12, 13, 21–23]. The application of non-in vitro culture techniques can circumvent some ethical and technical limitations. MSC enrichment technology, especially filtration enrichment, can lead to the direct adhesion of MSCs to the inner and outer surfaces of porous material by filtering bone marrow through porous material; this depends on the relatively strong adhesion of MSCs to achieve MSC screening, enrichment, and combination with biomaterials [14]. The filtration enrichment technique avoids any interference from exogenous agents and can be quickly completed during surgery, thus showing excellent clinical application prospects for cell therapy; its bone repair effects have been confirmed in animal experiments and certain clinical trials [13, 14, 24, 25]. This study explored the specific factors influencing the enrichment efficiency of stem cells, provided a theoretical basis for the clinical application of stem cell filtration enrichment, and evaluated the specific components of the filtered composite materials and the potential impact of the filtration procedure on stem cell safety.

### Specific factors influencing the enrichment efficiency of MSCs

When using the enrichment technique to prepare bioactive materials rich in MSCs for bone repair, a surgeon first selects bone substitutes of the appropriate type and volume according to the preoperative evaluation results, collects a certain volume of bone marrow to be filtered through the material multiple times, and finally implants the prepared composite material into the bone repair region. In this process, at least three elements need to be determined by the surgeon: the type of carrier material, the bone marrow aspiration volume (volume ratio of bone marrow/material), and the filtration frequency.

The type of carrier material used will directly influence the enrichment efficiency of the bone marrow cells because different types of materials have distinct three-dimensional structures, pore sizes, pore structures, surface morphology, and chemical composition. However, there are few reports on the enrichment efficiency of bone marrow when bone marrow is filtered through different carrier materials, even though this is of great significance for the effective integration of bone marrow active osteogenic components with porous bone substitute materials to promote bone defect repair. In this study, by considering the clinical utility and degradability of the materials, we found that three commonly used representative porous degradable materials, gelatin sponges, allogeneic bone particles, and porous β-TCP particles, could extract more than 50% of MSCs from bone marrow. This indicates that there is a wide selection of materials available for filtration enrichment with good stem cell enrichment efficiency. Notably, the gelatin sponge could achieve a high enrichment efficiency for MSCs, probably because it was rich in the cell adhesion molecule RGD [26]. The gelatin sponge also showed a 68.0% ± 10.8% enrichment efficiency for bone marrow NCs. The results, which showed high cell enrichment efficiency and low cell selectivity, indicate that this material showed poor selectively for MSCs from the original bone marrow and was not the optimal filtration medium. In contrast, allogeneic bone particles and porous β-TCP particles sacrificed cell enrichment efficiency to a certain extent in exchange for higher cell selectivity. Since the three materials have significant differences in terms of pore characteristics and chemical composition, it is difficult to determine which characteristics of the material were the primary contributing factors. Nevertheless, collagen is undeniably more favorable for cell adhesion due to its improved protein adsorption.

For a given type and volume of material, a limited cell-loading capacity makes it impossible to completely filter and combine the bone marrow MSCs, which indicates that excessive bone marrow extraction will reduce the enrichment efficiency of the target cells. In addition, an increase in the bone marrow volume may also be accompanied by the dilution of peripheral blood. According to a study by Muschler et al. [27], the number of bone marrow cell colonies was reduced by 50% when the volume of bone marrow aspirates from a single site increased from 1 to 4 ml. To maximize the number of bone marrow cells combined with materials while reducing the dilution of peripheral blood, we collected blood at a low volume and at multiple sites (2–4 ml/site). We used bone marrow suspensions with volumes that were two-, five-, and sevenfold the volume of the carrier material to evaluate the enrichment efficiency of the cells and to select the bone marrow aspirates with amounts that could effectively ensure the enrichment efficiency. When porous β-TCP particles were used as the filtration carrier material to filter bone marrow at a marrow/material volume ratio of 7:1, the enrichment efficiency was only 40.8% ± 15.9%, which was significantly lower than that of the twofold volume group. This suggested that the bone marrow volume in this case significantly surpassed the cell loading capacity of the carrier material. Nevertheless, the number of individual bone marrow cells is affected by factors such as gender, age, smoking history, and underlying diseases, and the bone marrow cell content can vary from one individual to another [28, 29]. Although filtration at a marrow/material volume of 2:1 could achieve satisfactory cell enrichment efficiency, it was difficult to ensure effective bone repair in some patients given the limited number of osteogenic progenitor cells in bone marrow with a volume that was twofold that of the material. Therefore, bone marrow with a volume fivefold that of the material may be more reliable in ensuring the number of enriched MSCs, and different carrier materials may require that the ratio be adjusted appropriately depending on the cell loading capacity of the material. It is noteworthy that the MSC enrichment efficiency (61.4% ± 5.4%) corresponding to an absolute volume of 5 ml of porous TCP particles was slightly higher compared to that corresponding to an absolute volume of 2 ml of porous TCP particles (55.6% ± 14.2%), despite the fact that both were used for filtration of the bone marrow/carrier material at a volume ratio of 5:1. The aggregation of particle materials produced during particle interactions due to an increase in material provided more possibilities for cell adhesion, thereby improving the enrichment efficiency of bone marrow MSCs. In clinical practice, the volume of material that can be implanted in the defect area often exceeds 5 ml, and the actual enrichment efficiency may increase further.

An increase in the number of filtrations actually means more chances of contact between the material and filtered bone marrow. In this study, the difference in bone marrow cell number among individuals resulted in a large difference in the bone marrow cell enrichment efficiency. Overall, 20 ml of bone marrow was filtered through 4 ml of porous β-TCP particles 5–6 times, and the enrichment efficiency basically stabilized. Thus, excessive filtration was not beneficial to the improvement of the enrichment efficiency but may exert a certain impact on cell activity. In clinical applications, due to the presence of uncontrollable factors, including individual characteristics, the filtration frequency is recommended to be no less than 5–6 to ensure the sufficient enrichment of bone marrow cells.

### Main cell components and their relatively quantitative distribution in the composite material after filtration

Filtration was used to combine the MSCs in the bone marrow with the carrier material to enhance the osteogenetic ability of the composite material. However, other cells in the bone marrow also remain in the material. Thus, a new cellular ecology that includes various cellular components is formed in the postfiltration composite. In comparison with the original bone marrow cell components, each of the MSCs in the composite material corresponds to fewer RBCs and WBCs, and MSCs have the predominant effect on the cell enrichment efficiency in the material. This suggests that the filtration process has a certain selectivity for MSCs. Many reports have indicated that MSCs show greater adherence compared with other NCs, but there are presently no clear explanations regarding the underlying mechanism [13, 30]. We believe for MSCs, cell size, cell morphology, and the expression of cell surface adhesion-associated proteins may be important factors for their adhesion. For PLs, the relatively high enrichment efficiency may be attributed to a certain degree of activation of PLs during repeated filtration (Fig. 3b). In addition, a cellular environment rich in MSCs and PLs is advantageous for bone repair and local wound healing.

In addition to cellular components, high-throughput antibody microarrays revealed a wide range of biologically active factors (such as VCAM-1, SDF-1α, EGF, angiopoietin-1/2, and IL family) in bone marrow that mediate biological processes, including cell adhesion, migration, proliferation, vascularization, and inflammatory responses. During filtration with the porous β-TCP particles, some of the active factors were absorbed by the carrier material. In fact, of all 105 active factors tested, only 15 active factors were absorbed by more than half of their total amount, and most soluble proteins could not be filtered or enriched. This suggested that the filtration enrichment technique had a good screening enrichment effect on MSCs and other components with a certain morphology but could not be used to enrich cytokines. The bone marrow pool, however, is a source that contains an abundance of cytokines [31]. Some of the active factors absorbed by the carrier materials may also have a positive effect on tissue repair.

### Influence of the filtration process on cell safety

The enrichment of bone marrow MSCs by autologous bone marrow filtration, followed by reimplantation, is highly safe, as it greatly avoids the interference of exogenous reagents on in vitro cultured cells and dramatic changes in the physical and chemical factors in the cell environment. In fact, the only factor that may potentially impact the cell is the filtration procedure itself. For the safety assessment of stem cell-based products, the Food and Drug Administration recommends a multiparameter test to analyze their characteristics that includes a cell morphology assessment, the detection of phenotype-specific cell surface antigens, a unique biochemical marker evaluation, and genomics analysis [32]. In this study, we compared the aspect ratios and osteogenic differentiation fates of bone marrow MSCs before and after filtration, and we did not observe any significant changes in either of these factors. In the same culture conditions, the bone marrow MSCs before and after filtration were highly consistent in terms of the cell cycle and apoptosis, which are two important cell life activities, and the expression of specific cell surface antigens. The gene expression profiles of MSCs before and after filtration were highly similar, and genes highly associated with high oncogenicity were not identified among the differentially expressed genes. These results suggested that the filtration operation itself had no negative impact on MSC safety.

Filtration itself does not affect cell safety and is the first step in cell therapy. In clinical settings, the implementation of enrichment techniques needs to meet stringent asepsis and other cell therapy requirements. Accordingly, some novel apparatuses have been developed to promote the clinical application of such techniques. George Frederick Muschler designed a detachable filtration device that can be placed on a sterile stand for MSC enrichment during surgery after all components of the apparatus are sterilized [33]. Yaokai Gan invented a system named SECCS that effectively isolates reimplanted biomaterials from outside contaminants through closed-loop pipes and double-layer filter boxes [13, 14]. In addition to ensuring safety, including asepsis, another key requirement of the current FDA regulations for cell therapy products is preclinical and clinical efficacy [34]. In fact, there have been several reports about the effectiveness of enrichment techniques for bone repair. Notably, Hernigou et al. [35, 36] noted that the number of enriched MSCs is a key factor in determining the repair effect. In this study, the analysis of factors influencing enrichment efficacy provides some basic guidance for achieving the full use of bone marrow MSCs.

## Conclusion

The MSCs in autologous bone marrow can be rapidly filtered and enriched in the carrier material by repeated filtration of autologous bone marrow through porous bone substitutes. The enrichment efficiency of MSCs can be optimized by selecting a suitable type of carrier material, adjusting the bone marrow/carrier material volume ratio and increasing the filtration frequency. The porous composite materials rich in MSCs that were formed after bone marrow filtration featured a cell ecology that was more favorable to osteogenesis. The filtration process did not significantly impact the safety of bone marrow MSCs. Therefore, the filtration of autologous bone marrow through porous bone substitutes can be used as a safe and effective method for the rapid preparation of MSC-based bone repair materials.

## Data Availability

All data generated or analysed during this study are included in this published article.

## Abbreviations

MSCs: mesenchymal stem cells
β-TCP: β-tricalcium phosphate
NCs: nucleated cells
RBCs: red blood cells
WBCs: white blood cells
PLs: platelets
CFU/ALP+: alkaline phosphatase-positive colony-forming units
